# Design, synthesis and biological activity evaluation of a new class of 2,4-thiazolidinedione compounds as insulin enhancers

**DOI:** 10.1080/14756366.2019.1608197

**Published:** 2019-05-10

**Authors:** Zou Huiying, Chen Guangying, Zhou Shiyang

**Affiliations:** a College of Chemistry and Chemical Engineering, Hainan Normal University, Haikou, China;; b Key Laboratory of Tropical Medicinal Resource Chemistry of Ministry of Education, Hainan Normal University, Haikou, China

**Keywords:** Diabetes mellitus, insulin enhancers, design, synthesis, 2,4-thiazolanedione

## Abstract

Diabetes mellitus (DM) is a global disease with a high incidence of type 2 diabetes. Current studies have shown that insulin enhancers play an important role in the treatment of type 2 diabetes and have great importance in the improvement of type 2 diabetes. In this research, Rosiglitazone was taken as the lead compound, and the structure was modified by using the bioisostere principle, and a new class of 2,4-thiazolanedione compound was designed and synthesised. The novel series of compounds were studied for their biological activities *in vitro* and *in vivo*. *In vitro* tests, the biological activities showed that the target compounds have good selective activation of peroxisome-proliferator-activated receptor γ (PPARγ), such as the compounds **6a**, **6e**, **6f**, **6g** and **6i**, especially the compound **6e** to PPARγ was EC_50_ = 0.03 ± 0.01 μmol/L *in vitro*. Then, *in vivo* biological activities’ test results showed that the tendency of increasing in blood sugar had an obvious inhibiting effect, and had a significant insulin hypoglycaemic effect of enhancing and extending the exogenous. In addition, the results of cytotoxicity tests and acute toxicity tests (LD_50_) showed that these compounds belong to the low toxicity compounds.

## 1. Introduction

**Figure 1.**
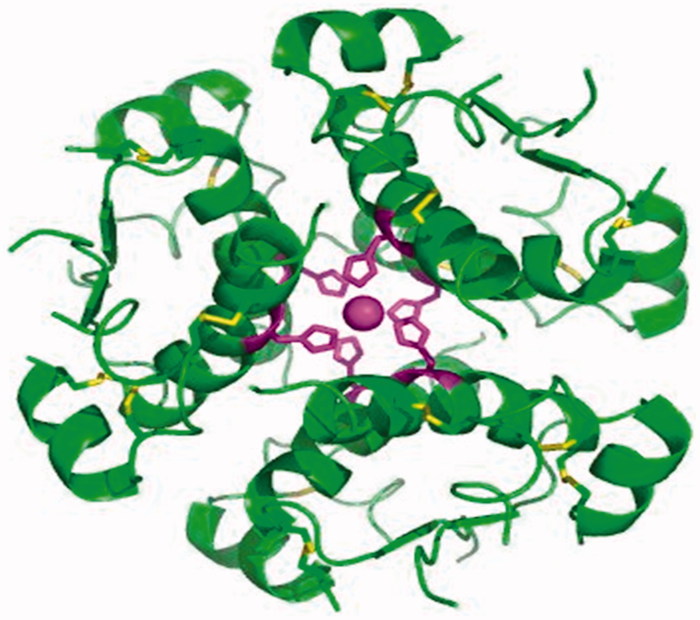
The protein structure of insulin^20–22^.

**Figure 2.**
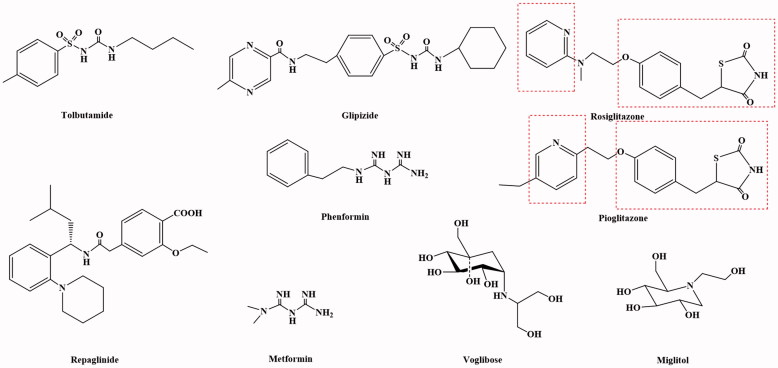
The structure of NIDDM drugs.

Diabetes mellitus (DM) is a kind of disease with carbohydrate, protein and fat metabolism disorder caused by multiple aetiologies, which has a high incidence, only after those of cardiovascular disease and tumour^1–3^. Based on the 16th International Diabetes Federation (IDF) conference in 1997, diabetes could be divided into two broad categories: insulin-dependent diabetes mellitus (IDDM, I diabetes) and non-insulin-dependent diabetes mellitus (NIDDM, 2 diabetes)^4–8^. The IDDM patients of pancreatic islet β-cells in the body were damaged^9–14^, the level of insulin in the blood plasma was far lower than the normal value, and most of the patients were under 30 years old^15–19^. The disease (1) was mainly treated with insulin^20–22^ (Figure 1) and its analogues^23–25^. The NIDDM patients had less insulin resistance and secretion disorder, normal or slightly lower insulin level in plasma, and the internal target tissue of the body was not sensitive to insulin response^26–30^. Due to the relative insufficiency of insulin, hyperglycaemia caused, and most of the patients were over 40 years old. Insulin resistance, especially in the liver and muscle tissue insulin resistance of type 3, plays a very important role in the pathogenesis of diabetes^31–33^. The NIDDM patients account for about more than 90% of the patients, and the main drugs used for treatment were promoter to insulin secretion, insulin enhancers and α-glucosidase inhibitors^34–36^ (Figure 2). In addition to the main drugs delivery was injection way of insulin, the other was used oral way for all kinds of type 2 diabetes drugs, so it was also called oral medications.

**Figure 3.**
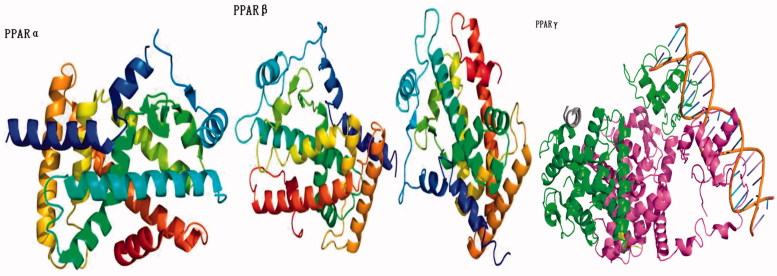
The protein structure of PPAR^52–58^.

In recent years, the research has shown that the occurrence and development of insulin resistance in type 2 plays an extremely important role. The main reason for insulin resistance was that the binding of insulin antibodies and insulin inhibits the transport of insulin targets, the insulin receptors on the target cells were reduced in hyperinsulinaemia, and the acidosis made the body to become less sensitive to insulin. Therefore, the development and use of drugs that could improve patients' insulin sensitivity and improve the state of insulin resistance were of great significance for the treatment of diabetes. The insulin enhancers used clinically include biguanides (Metformin^37^ and Phenformin^38–42^) and thiazolidinediones (Rosiglitazone^43–45^ and Pioglitazone^46–51^) (Figure 2). At present, thiazolidinedione hypoglycaemic drugs were the main type of insulin enhancers. These drugs have all characteristics of thiazolidinedione in terms of chemical structure and could also be regarded as derivatives of phenylpropionic acid. The mechanism of action of these drugs could increase the sensitivity of insulin to the target tissue of the receptor, reduce the production of liver sugars and enhance the glucose uptake in peripheral tissues. The target of its action was the peroxidase-proliferator activated receptor (PPAR) of nucleus^52–58^. The PPAR had three types: PPARα, PPARβ and PPARγ (Figure 3). The thiazolidinediones drug could be activated to PPARγ, increase the sensitivity of fat cells, liver cells and skeletal muscle cells to insulin, and promote the uptake, transport and oxidation of insulin target cells to blood sugar. At the same time, it can reduce the content of blood sugar and free fatty acids. In addition, Rosiglitazone also increases glucose uptake by glucose transporters 1 and 4. Rosiglitazone could also improve atherosclerosis and correct lipid disorders. The main adverse effects of Rosiglitazone were elevated liver transaminase levels, mild oedema and anaemia^59^.

**Figure 4.**
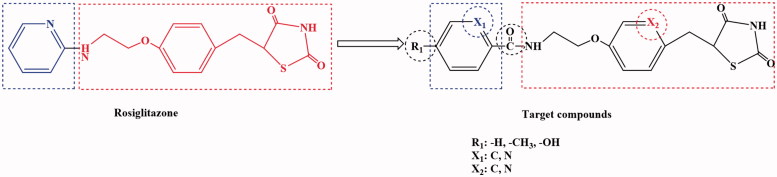
Design of 2,4-thiazolidinedione compounds.

**Scheme 1.**
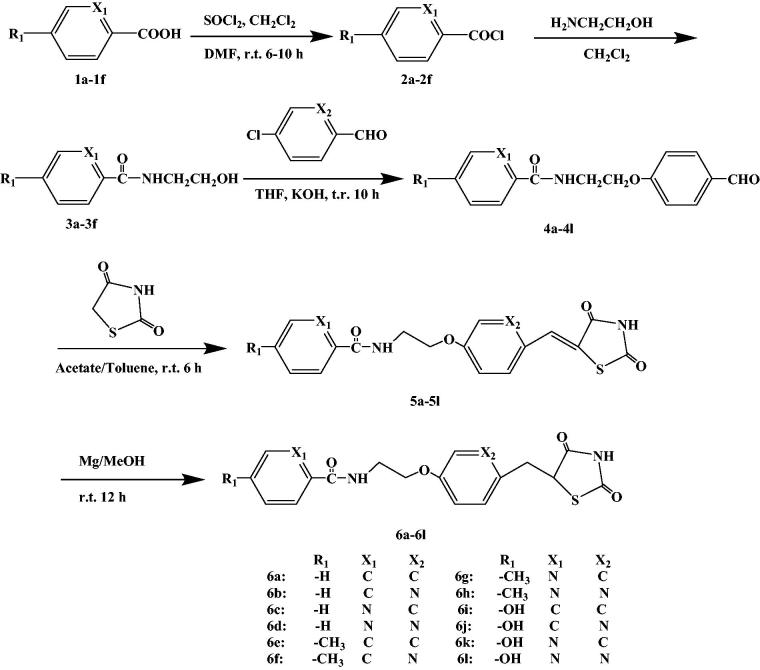
The synthetic route of 2,4-thiazolidinedione compounds.

By combining the advantages and disadvantages of thiazolidinediones drug in the clinical application, a new type of thiazolidinedione structure was designed with Rosiglitazone as the lead compound and modified by the bioisostere principle (Figure 4). The newly designed target compounds retain the basic skeleton structure of Rosiglitazone from a chemical perspective, and mainly optimise the structure of benzene ring and its substituents to achieve the design of compounds with good biological activity, low toxic and side effects. These target compounds were synthesised by five steps of acylchlorination, amidation, condensation, knoevenagel and addition reaction (Scheme 1). The synthetic route was characterised by simple operation, high total yield and mild reaction conditions.

## 2. Results and discussion

### 2.1. The design and synthesis of target compounds

Rosiglitazone was a thiazolanedione hypoglycaemic agent, which belongs to insulin enhancers that were used to treat type 2 diabetes. A recent study^43–45^ showed that Rosiglitazone has a mechanism of action of PPARγ and increases glucose uptake by glucose transporters 1 and 4. The Rosiglitazone could also improve atherosclerosis and correct lipid disorders. The main adverse effects of Rosiglitazone were elevated liver transaminase levels, mild oedema and anaemia. It was against these shortcomings, the Rosiglitazone as the lead compound, designed and synthesised of a new class of 2,4-thiazolanedione compounds for the treatment of type 2 diabetes which was very necessary. The novel series of compounds were designed, and retain the basic skeleton of Rosiglitazone in terms of its chemical structure, namely 2,4-thiazolidinedione and six-membered ring structure (Figure 4). According to the structure–activity relationship (SAR), the target compounds should have the same treatment effect as Rosiglitazone or be superior to its effect. In the design of the target compounds, the six-membered ring and substituents on the ring were modified. The specific structure modification was selection C atom and N atom on the six-membered ring by using the modified bioisostere principle, and the substituents on the ring selection –H, –CH_3_ and –OH. Such structural modification was designed to change the log*P*, p*Ka* and spatial structures of the target compounds in order to obtain the target compounds with good biological activity and little toxic and side effects. In terms of synthetic route, we selected a synthetic route with simple operation, mild reaction conditions and high total yield. The synthesis of the target compounds was of five steps: acylchlorination, amidation, condensation, knoevenagel and addition reaction (Scheme 1). A total of 12 compounds were synthesised and they were analysed by ^1^H NMR, 13C NMR, MS and elemental analysis. The yield of the target compounds **6a**–**6f** was 69.8–84.2%.

### 2.2. The biological activities

#### 2.2.1. The biological activity screening *in vitro*

**Figure 5.**
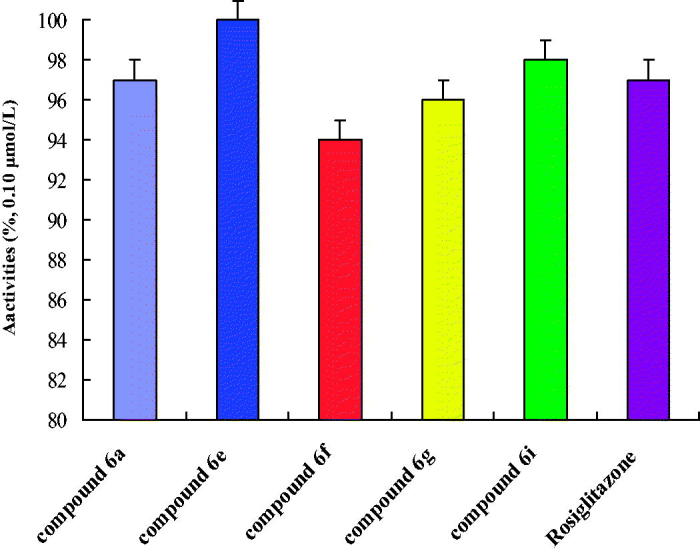
*In vitro* activities of PPARγ agonists activity.

Drug *in vitro* experiment was one of the basic contents to evaluate drugs activity, and it was a necessary approach to research drugs. Drugs’ efficacy was often affected by some physical and chemical properties of compounds, such as lipid–water partition coefficient (log *p*), and dissociation degree (p*Ka*). In this part of the research, the physical and chemical properties of 2,4-thiazolidinedione compounds were studied that were very necessary to study the pharmacodynamics (Table 1). In Table 1, we could find that target compounds were fat-soluble. The fat-soluble compounds were favourable for drugs absorption, and drugs were easy to enter the cells. This compound the log *p* was from 1.45 to 3.26. The p*Ka* will affect the absorption and delivery of the drugs, different environmental (acid and alkalinity) medicine may be different, sometimes exists in the form of ions, sometimes exists in the form of molecules. Usually in the form of ion drugs has no effect. Therefore, it was necessary to study the dissociation degree (p*Ka*) of drugs to study the pharmacodynamics. In Table 1, we could find that target compounds the p*K_a_* were from 8.58 to 10.43, and these compounds could be absorbed in the intestinal tract. After the physical and chemical properties were studied, the biological activity was studied. The *in vitro* biological activity evaluation was based on the concentration of 50% of maximal effect (EC_50_), and PPARα, PPARβ and PPARγ were selected (Table 1). In Table 1, we can find that these compounds had a good biological activity to PPARγ, but inactive to PPARα and PPARβ. These data show that these kinds of target compounds have good selectivity. Especially, as compounds **6a**, **6e**, **6f**, **6g** and **6i** had better biological activity *in vitro*, which of compound **6e** to PPARγ was EC_50_ = 0.03 ± 0.01 μmol/l. The *in vitro* biological activity evaluation was based on the concentration of 50% of maximal effect (EC_50_), and PPARα, PPARβ and PPARγ were selected (Table 1). In Table 1, we can found that these compounds had a good biological activity to PPARγ, but inactive to PPARα and PPARβ. These data showed that these kinds of target compounds have good selectivity. Especially, as compounds **6a**, **6e**, **6f**, **6g** and **6i** had better biological activity *in vitro*, which of compound **6e** to PPARγ was EC_50_ = 0.03 ± 0.01 μmol/l. After that, the target compounds **6a**, **6e**, **6f**, **6g** and **6i** had good biological activity and were preliminarily screened out for further study on PPARγ receptor agonist activity (Figure 5). The experimental results showed that the five target compounds had good biological activity *in vitro*. According to the structure–activity relationship (SAR), different R_1_ (–H, –CH_3_ and –OH) substituents have little effect on EC_50_, which was mainly affected by the two six-membered ring structures in the target compounds. As could be seen from the data of *in vitro* experiments, when both six-membered rings were benzene rings, the target compounds had a good biological activity to PPARγ. In addition, when the first six-membered ring was pyridine ring and the second six-membered ring was benzene ring, it also shows a good biological activity to PPARγ.

**Table 1.** I*n vitro* the activation of test compounds on PPARα, PPARβ and PPARγ.

Compounds	R_1_	X_1_	X_2_	log *p*	p*K_a_*	EC_50_ ± *SD*^a^（μmol/l）		
						PPARα	PPARβ	PPARγ
**6a**	–H	C	C	2.68	10.43	ia^b^	ia	0.08 ± 0.02
**6b**	–H	C	N	1.77	10.38	12.90 ± 1.29	9.18 ± 0.89	1.29 ± 0.53
**6c**	–H	N	C	1.92	10.00	13.02 ± 1.56	ia	0.45 ± 0.21
**6d**	–H	N	N	1.38	9.78	ia	12.19 ± 1.21	3.22 ± 0.65
**6e**	–CH_3_	C	C	3.26	9.77	ia	ia	0.03 ± 0.01
**6f**	–CH_3_	C	N	2.56	9.65	ia	ia	0.10 ± 0.10
**6g**	–CH_3_	N	C	2.83	9.45	ia	ia	0.08 ± 0.03
**6h**	–CH_3_	N	N	2.01	9.41	10.56 ± 1.86	ia	0.28 ± 0.13
**6i**	–OH	C	C	2.80	9.03	ia	ia	0.07 ± 0.02
**6j**	–OH	C	N	2.11	9.01	ia	14.53 ± 1.20	0.34 ± 0.15
**6k**	–OH	N	C	2.32	8.66	ia	ia	0.21 ± 0.11
**6l**	–OH	N	N	1.45	8.58	9.34 ± 1.01	10.11 ± 1.12	2.68 ± 0.46
Rosiglitazone	/	/	/	/	/	9.05 ± 1.15	12.23 ± 1.35	0.08 ± 0.02
DMSO	/	/	/	/	/	ia	ia	ia

a Data represent the mean values from at least eight independent experiments each in triplicate (*n* ≥ 8).

b ia = inactive.

#### 2.2.2. The evaluation of biological activity *in vivo*

The compounds **6a**, **6e**, **6f**, **6g** and **6i** were selected from the *in vivo* activity test, and it was used for *in vivo* activity evaluation. *In vivo* experiments, glucose tolerance test, insulin tolerance test, cell survival experiment and acute toxicity were used as the test indicators (Tables 2, 3 and 4). In Table 2, we chose dose 10 mg kg^−1^ d^−1^ and used oral administration. The Rosiglitazone was used as the positive reference substance and DMSO was used as the blank control. The mice were given the drug for 10 d, blood was collected from the tail vein of the mice before and after the sugar load for 1 h and 2 h, and the blood glucose level of the mice before and after the sugar load was measured by the glucometer. The experimental results showed that the compounds **6a**, **6e**, **6f**, **6g** and **6i** had good resistance activation to glucose. In Table 3, experimental results showed that the compounds **6a**, **6e**, **6f**, **6g** and **6i** had good resistance activation to insulin. In Table 4, the results of cell survival experiments show that in the compounds **6a**, **6e**, **6f**, **6g** and **6i** in the 10^−3^–10^−2 ^mol L^−1^, no obvious cytotoxic effect was observed. In Table 4, the acute toxicity test results show the oral administration of the compounds, and it belongs to low toxicity compounds.

**Table 2.** The oral glucose tolerance test.

Compounds	Dose (mg kg^−1^ d^−1^)	Blood glucose levels^a^ (mmol l^−1^)		
		0 h	1 h	2 h
**6a**	10	3.376 ± 0.361	6.342 ± 2.011	3.368 ± 1.341
**6e**	10	3.312 ± 0.301	6.003 ± 1.876	2.953 ± 0.876
**6f**	10	3.216 ± 0.315	6.651 ± 2.413	4.121 ± 1.922
**6g**	10	3.112 ± 0.279	6.445 ± 2.220	3.677 ± 1.730
**6i**	10	3.218 ± 0.317	6.401 ± 2.112	3.265 ± 1.236
Rosiglitazone	10	3.265 ± 0.345	6.351 ± 2.004	3.654 ± 1.232
DMSO	10	3.471 ± 0.211	8.756 ± 2.102	4.371 ± 1.621

a Data represent the mean values from at least 10 independent experiments each in triplicate (*n* ≥ 10).

**Table 3.** The effect of compounds on blood glucose under insulin load.

Compounds	Dose (mg kg^−1^ d^−1^)	Blood glucose levels^a^ (mmol l^−1^)		
		0 h	1 h	2 h
**6a**	10	5.644 ± 1.035	1.801 ± 0.531	2.498 ± 1.136
**6e**	10	5.411 ± 1.002	1.762 ± 0.453	2.463 ± 1.211
**6f**	10	5.714 ± 1.465	1.903 ± 0.611	2.602 ± 1.143
**6g**	10	5.613 ± 1.246	1.843 ± 0.544	2.566 ± 1.224
**6i**	10	5.592 ± 1.134	1.792 ± 0.541	2.501 ± 1.025
Rosiglitazone	10	5.613 ± 1.124	1.811 ± 0.497	2.513 ± 1.072
DMSO	10	5.214 ± 0.761	1.842 ± 0.513	3.024 ± 1.671

a Data represent the mean values from at least 10 independent experiments each in triplicate (*n* ≥ 10).

**Table 4.** The cytotoxicity and acute toxicity tests.

Compounds	Cell survival rate^a^ (%)					LD_50_ ± *SD*^b^(mg kg^−1^)
	10^−6^ mol l^−1^	10^−5^ mol l^−1^	10^−4^ mol l^−1^	10^−3^ mol l^−1^	10^−2^ mol l^−1^	
**6a**	100	99.9 ± 0.11	99.3 ± 0.26	98.1 ± 0.28	93.1 ± 1.21	2313.6 ± 1.26
**6e**	100	100	100	99.1 ± 0.08	94.3 ± 1.02	2451.2 ± 1.24
**6f**	100	99.8 ± 0.09	99.2 ± 0.31	98.3 ± 0.45	89.6 ± 1.34	2013.6 ± 1.43
**6g**	100	100	99.8 ± 0.09	98.6 ± 0.44	90.5 ± 1.54	2138.8 ± 1.36
**6i**	100	100	99.9 ± 0.02	98.8 ± 0.29	92.1 ± 1.43	2268.9 ± 1.25
Rosiglitazone	99.8 ± 0.05	99.5 ± 0.21	98.1 ± 0.61	97.2 ± 0.71	86.5 ± 2.41	2246.1 ± 1.56

a Data represent the mean values from at least ten independent experiments each in triplicate (*n* ≥ 10).

b Data represent the mean values from at least three independent experiments each in triplicate (*n* ≥ 3).

## 3. Conclusion

We report here the design, synthesis and evaluation of a series of novel PPARγ selective activators containing 2,4-thiazolanedione compounds. Rosiglitazone is used as the lead compound, a total of 12 compounds (**6a–6l**) were designed and synthesised, and used the bioisostere principle to modify the lead compound. We selected five steps to synthesis the target compounds, and the route with high total yield, mild conditions and simple operation. Structure–activity relationship (SAR) studies led to the identification of PPARγ selective activators (the compounds **6a**, **6e**, **6f**, **6g** and **6i**) with stronger potency and efficacy to activate PPARγ. The compound **6e** to PPARγ was EC_50_ = 0.03 ± 0.01 μmol/L *in vitro.* The *in vivo* biological activity test results showed that the tendency of increasing the blood sugar had obvious inhibition, and could significantly enhance and extend the exogenous insulin hypoglycaemic effect. In addition, the results of cytotoxicity test and acute toxicity test showed that these compounds belong to low toxicity compounds.

## 4. Experimental

### 4.1. Chemistry section

#### 4.1.1. A general method for synthesis of compounds 2a to 2f

The benzoic acid (**1a**, 12.03 g, 0.10 mol) was put into 500 ml round bottom flask, then 200 ml of methylene chloride (CH_2_Cl_2_) was used as the solvent and 10 drops of *N,N’*-dimethyl formamide (DMF) used as a catalyst for the reaction were added. The flask was placed in the ice water bath (≤10 °C), and a magnetic mixer was used to stir until the reaction liquid became clarified. The thionyl chloride (SOCl_2_, 11.25 ml, 0.15 mol) was constantly dropped into the flask under stirring, and the drop speed rate and the reaction temperature were controlled (≤10 °C and ≥20 min). After the reactants were added, the reaction lasted for 6–10 h under reflux. The methylene chloride and excessive thionyl chloride were removed under vacuum. The mixture was dried to get the crude product of benzoyl chloride (**2a**). The crude product was obtained by atmospheric distillation, the product was collected at 198 °C, and the pure product of benzoyl chloride (**2a**) as a colourless transparent liquid was obtained. The general method was used to synthesise compounds **2c** and **2e** as all were colourless transparent liquid. And the general method was used to synthesise crude compounds **2b**, **2d** and **2f**, the crude products were recrystallised with hexane, filtered and dried in vacuum to give pure products of compounds **2b**, **2d** and **2f** as all were of white crystal.

#### 4.1.2. A general method for synthesis of compounds 3a to 3f

The benzoyl chloride (**2a**, 14.05 g, 0.10 mol) was put into 500 ml round bottom flask, then 200 ml of methylene chloride (CH_2_Cl_2_) was added as a solvent for the reaction. The flask was placed in the ice water bath (0–10 °C), and a magnetic mixer was used to stir until the reaction liquid became clarified. The ethanolamine (6.11 g, 0.10 mol) was constantly dropped into the flask under stirring, and the drop speed rate and the reaction temperature were controlled (0–10 °C and ≥20 min). After the reactants were added, the reaction lasted 6 h under standing. The methylene chloride (CH_2_Cl_2_) was removed under vacuum. The mixture was dried to get the crude product of *N*-(2-hydroxyethyl) benzamide (**3a**). The crude product was recrystallised with toluene, filtered and dried in vacuum to give pure product of 4-chloro-*N*-(hydroxymethyl) benzamide (**3a**) as a white crystal. The general method was used to synthesis compound **3b** to **3f** as all were of white crystal.

#### 4.1.3. A general method for synthesis of compounds 4a to 4l

The compound **3a** (16.51 g, 0.10 mol) and the solid potassium hydroxide (5.61 g, 0.01 mol) were put into 500 ml round bottom flask, and 100 ml tetrahydrofuran (THF) was added as the reaction solvent. Under room temperature with magnetic stirring, the 4-chlorobenzaldehyde (14.05 g, 0.10 mol) was added at a constant pressure by a drop funnel, and the rate of droplet acceleration was controlled. When the dripping was completed, under the condition of nitrogen protection, the reaction was refluxed for 10 h. When the reaction was complete, the reaction solution was poured into the ice water, then the ethyl acetate extract was added three times, and added with the superstratum extract. The extract was washed twice with saturated sodium chloride solution and dried with anhydrous calcium chloride. Decompression distillation and recovery of ethyl acetate were done to obtain the crude product of compound **4a**. The crude product was recrystallized with toluene filtered, and dried in vacuum to give pure product of compound **4a** with a white crystal. The general method was used to synthesise compounds **4b** to **4l** as all were of white crystal.

#### 4.1.4. A general method for synthesis of compounds 5a to 5l

The compound **4a** (26.93 g, 0.10 mol) and 2,4-thiazolanedione (11.71 g, 0.10 mol) were put into 500 ml round bottom flask. The 100 ml of glacial acetic acid was added as the reaction solvent. Ten drops of hexahydropyridine were added as the reaction catalyst, and then 30 ml of toluene was added as the water solvent. The water divider was connected that divided water for 6 h. When the water was separated, the reaction liquid was cooled and then refrigerated for 24 h. The precipitate was precipitated, filtered and the precipitate was washed with ether for three times, and the mixture was vacuum dried to get the crude product of compound **5a**. The crude product was recrystallised with toluene to give pure product of compound **5a** which is of a white crystal. The general method was used to synthesise compounds **5b** to **5l** as all were of white crystal.

#### 4.1.5. A general method for synthesis of compounds 6a to 6l

The compound **5a** (37.04 g, 0.10 mol) was put into 500 ml round bottom flask, then 200 ml of methanol was added as the solvent. The magnesium powder (24.00 g, 0.10 mol) was added to a round bottom flask, and the refluxing reaction was performed for 12 h with the protection of nitrogen. After the reflux reaction was completed, it was immediately filtrated, the filtrate was collected, and the filtrate was poured into the ice water. The extract was extracted three times with ethyl acetate, combined with the superstratum extract, and washed twice with saturated sodium bicarbonate and saturated sodium chloride solution, and dried with anhydrous calcium chloride after washing. Decompression distillation and recovery of solvent methanol were done to obtain the crude product of compound **5a**. The crude product was recrystallized with methanol to give pure product of compound **6a** which is of a white powder. The general method was used to synthesise compounds **6b** to **6l** as all were of white powder.

*N*-(2–(4-((2,4-dioxothiazolidin-5-yl) methyl) phenoxy)ethyl) benzamide (**6a**): white powder, yield 84.2%, m.p. 164–166 °C; ^1^H NMR (300 MHz, DMSO) *δ*: 3.32 (2H, d, *J* = 6.1 Hz, –CH_2_–), 3.48 (2H, t, *J* = 7.2 Hz, –CH_2_–), 4.14 (2H, t, *J* = 7.2 Hz, –CH_2_–), 4.46 (1H, t, *J* = 6.1 Hz, –CH–), 6.92 (2H, ddd, *J* = 8.8, 1.8, 0.5 Hz, Ph-H), 7.11 (2H, ddd, *J* = 8.8, 1.0, 0.5 Hz, Ph-H), 7.48 (2H, dddd, *J* = 8.5, 7.5, 1.4, 0.4 Hz, Ph-H), 7.63 (1H, tt, *J* = 7.5, 1.5 Hz, Ph-H), 7.85 (2H, dddd, *J* = 8.5, 1.6, 1.5, 0.4 Hz, Ph-H); 13C NMR (75 MHz, DMSO) *δ*: 39.4, 39.7, 49.8, 61.2, 115.2, 127.6, 127.9, 128.5, 128.9, 133.7, 1, 158.4, 168.1, 169.1, 175.8; HR-ESI-MS m/z: calcd for C_19_H_18_N_2_O_4_S｛(M + H)^+^｝ 370.0986, found 370.4231; Anal. calcd for C_19_H_18_N_2_O_4_S: C, 61.61; H, 4.90; N, 7.56; O, 17.28; S, 8.65; found: C, 61.62; H, 4.91; N, 7.54; O, 17.27; S, 8.66%.

*N*-(2-((6-((2,4-dioxothiazolidin-5-yl) methyl) pyridin-3-yl) oxy) ethyl) benzamide (**6b**): white powder, yield 82.7%, m.p. 171–173 °C; ^1^H NMR (300 MHz, DMSO) *δ*: 3.14 (2H, d, *J* = 6.3 Hz, –CH_2_–), 3.48 (2H, t, *J* = 6.5 Hz, –CH_2_–), 4.15 (2H, t, *J* = 6.5 Hz, –CH_2_–), 4.46 (1H, t, *J* = 6.3 Hz, –CH–), 7.03 (1H, dd, *J* = 8.0, 0.5 Hz, Py-H), 7.32 (1H, dd, *J* = 8.0, 1.6 Hz, Py-H), 7.46 (2H, dddd, *J* = 8.5, 7.5, 1.4, 0.4 Hz, Ph-H), 7.65 (1H, tt, *J* = 7.5, 1.5 Hz, Ph-H), 7.78 (2H, dddd, *J* = 8.5, 1.6, 1.5, 0.4 Hz, Ph-H), 8.23 (1H, dd, *J* = 1.6, 0.5 Hz, Py-H); 13C NMR (75 MHz, DMSO) *δ*: 30.4, 39.4, 49.8, 61.2, 122.1, 127.8, 128.6, 129.7, 133.7, 142.6, 151.4, 160.4, 168.1, 169.2, 175.7; HR-ESI-MS m/z: calcd for C_18_H_17_N_3_O_4_S｛(M + H)^+^｝ 371.0943, found 371.4112; Anal. calcd for C_18_H_17_N_3_O_4_S: C, 58.21; H, 4.61; N, 11.31; O, 17.23; S, 8.63; found: C, 58.22; H, 4.60; N, 11.32; O, 17.21; S, 8.64%.

*N*-(2-(4-((2,4-dioxothiazolidin-5-yl) methyl) phenoxy) ethyl) picolinamide (**6c**): white powder, yield 80.5%, m.p. 174–176 °C; ^1^H NMR (300 MHz, DMSO) *δ*: 2.88 (2H, d, *J* = 6.1 Hz, –CH_2_–), 3.50 (2H, t, *J* = 7.1 Hz, –CH_2_–), 4.17 (2H, t, *J* = 7.1 Hz, –CH_2_–), 4.49 (1H, t, *J* = 6.1 Hz, –CH–), 7.01 (2H, ddd, *J* = 8.8, 1.8, 0.5 Hz, Ph-H), 7.12 (2H, ddd, *J* = 8.8, 1.0, 0.5 Hz, Ph-H), 7.56 (1H, ddd, *J* = 7.6, 5.2, 1.7 Hz, Py-H), 7.94–8.02 (2H, 8.01 (ddd, *J* = 8.2, 1.7, 0.5 Hz, Py-H), 7.97 (ddd, *J* = 8.2, 7.6, 1.9 Hz, Py-H)), 8.71 (1H, ddd, *J* = 5.2, 1.9, 0.5 Hz, Py-H); 13C NMR (75 MHz, DMSO) *δ*: 39.2, 39.7, 49.8, 60.8, 115.2, 122.6, 123.4, 127.7, 134.0, 137.2, 148.3, 151.7, 158.4, 164.5, 169.0, 175.6; HR-ESI-MS m/z: calcd for C_18_H_17_N_3_O_4_S｛(M + H)^+^｝ 371.0942, found 371.4111; Anal. calcd for C_18_H_17_N_3_O_4_S: C, 58.21; H, 4.61; N, 11.31; O, 17.23; S, 8.63; found: C, 58.22; H, 4.60; N, 11.33; O, 17.22; S, 8.62%.

*N*-(2-((6-((2,4-dioxothiazolidin-5-yl) methyl) pyridin-3-yl) oxy) ethyl) picolinamide (**6d**): white powder, yield 73.9%, m.p. 189–191 °C; ^1^H NMR (300 MHz, DMSO) *δ*: 3.12 (2H, d, *J* = 6.3 Hz, –CH_2_–), 3.49 (2H, t, *J* = 6.9 Hz, –CH_2_–), 4.18 (2H, t, *J* = 6.9 Hz, –CH_2_–), 4.42 (1H, t, *J* = 6.3 Hz, –CH–), 7.02 (1H, dd, *J* = 8.0, 0.5 Hz, Py-H), 7.33 (1H, dd, *J* = 8.0, 1.6 Hz, Py-H), 7.57 (1H, ddd, *J* = 7.6, 5.2, 1.7 Hz, Py-H), 7.92–8.02 (2H, 8.01 (ddd, *J* = 8.2, 1.7, 0.5 Hz, Py-H), 7.97 (ddd, *J* = 8.2, 7.6, 1.9 Hz, Py-H)), 8.27 (1H, dd, *J* = 1.6, 0.5 Hz, Py-H), 8.70 (1H, ddd, *J* = 5.2, 1.9, 0.5 Hz, Py-H); 13C NMR (75 MHz, DMSO) *δ*: 30.4, 39.2, 49.9, 60.8, 122.0, 122.6, 123.5, 129.7, 137.4, 142.6, 148.4, 151.4, 151.8, 160.4, 164.5, 169.0, 175.7; HR-ESI-MS m/z: calcd for C_17_H_16_N4O_4_S｛(M + H)^+^｝ 372.0891, found 372.3992; Anal. calcd for C_17_H_16_N4O_4_S: C, 54.83; H, 4.33; N, 15.05; O, 17.18; S, 8.61; found: C, 54.81; H, 4.34; N, 15.06; O, 17.17; S, 8.62%.

*N*-(2-(4-((2,4-dioxothiazolidin-5-yl) methyl) phenoxy) ethyl)-4-methylbenzamide (**6e**): white powder, yield 75.1%, m.p. 170–172 °C; ^1^H NMR (300 MHz, DMSO) *δ*: 2.32 (3H, s, –CH_3_), 2.88 (2H, d, *J* = 6.1 Hz, –CH_2_–), 3.48 (2H, t, *J* = 7.2 Hz, –CH_2_–), 4.15(2H, t, *J* = 7.2 Hz, –CH_2_–), 4.47 (1H, t, *J* = 6.1 Hz, –CH–), 7.01 (2H, ddd, *J* = 8.8, 1.8, 0.5 Hz, Ph-H), 7.06–7.20 (4H, 7.16 (ddd, *J* = 8.5, 1.2, 0.5 Hz, Ph-H), 7.11 (ddd, *J* = 8.8, 1.0, 0.5 Hz, Ph-H)), 7.86 (2H, ddd, *J* = 8.5, 1.7, 0.5 Hz, Ph-H); 13C NMR (75 MHz, DMSO) *δ*: 21.4, 39.2, 39.7, 49.8, 60.8, 115.2, 127.5, 127.9, 128.6, 131.0, 139.7, 158.5, 168.3, 169.0, 175.6; HR-ESI-MS m/z: calcd for C_20_H_20_N_2_O_4_S｛(M + H)^+^｝ 384.1142, found 384.4503; Anal. calcd for C_20_H_20_N_2_O_4_S: C, 62.48; H, 5.24; N, 7.29; O, 16.65; S, 8.34; found: C, 62.46; H, 5.25; N, 7.28; O, 16.66; S, 8.35%.

*N*-(2-((6-((2,4-dioxothiazolidin-5-yl) methyl) pyridin-3-yl) oxy) ethyl)-4-methylbenzamide (**6f**): white powder, yield 74.2%, m.p. 177–179 °C; ^1^H NMR (300 MHz, DMSO) *δ*: 2.32 (3H, s, –CH_3_), 3.12 (2H, d, *J* = 6.3 Hz, –CH_2_–), 3.48 (2H, t, *J* = 6.5 Hz, –CH_2_–), 4.18 (2H, t, *J* = 6.5 Hz, –CH_2_–), 4.42 (1H, t, *J* = 6.3 Hz, –CH–), 7.01 (1H, dd, *J* = 8.0, 0.5 Hz, Py-H), 7.16 (2H, ddd, *J* = 8.5, 1.2, 0.5 Hz, Ph-H), 7.35 (1H, dd, *J* = 8.0, 1.6 Hz, Py-H), 7.86 (2H, ddd, *J* = 8.5, 1.7, 0.5 Hz, Ph-H), 8.26 (1H, dd, *J* = 1.6, 0.5 Hz, Py-H); 13C NMR (75 MHz, DMSO) *δ*: 21.2, 30.6, 39.4, 49.8, 60.8, 122.0, 127.5, 128.6, 129.4, 131.0, 139.7, 142.6, 151.5, 160.2, 168.1, 169.0, 175.6; HR-ESI-MS m/z: calcd for C_19_H19N_3_O_4_S｛(M + H)^+^｝ 385.1094, found 385.4381; Anal. calcd for C_19_H19N_3_O_4_S: C, 59.21; H, 4.97; N, 10.90; O, 16.60; S, 8.32; found: C, 59.23; H, 4.96; N, 10.91; O, 16.60; S, 8.30%.

*N*-(2-(4-((2,4-dioxothiazolidin-5-yl) methyl) phenoxy) ethyl)-5-methylpicolinamide (**6g**): white powder, yield 73.1%, m.p. 180–182 °C; ^1^H NMR (300 MHz, DMSO) *δ*: 2.32 (3H, s, –CH_3_), 3.12 (2H, d, *J* = 6.3 Hz, –CH_2_–), 3.48 (2H, t, *J* = 6.5 Hz, –CH_2_–), 4.18(2H, t, *J* = 6.5 Hz, –CH_2_–), 4.42 (1H, t, *J* = 6.3 Hz, –CH–), 7.01 (1H, dd, *J* = 8.0, 0.5 Hz, Py-H), 7.16 (2H, ddd, *J* = 8.5, 1.2, 0.5 Hz, Ph-H), 7.35 (1H, dd, *J* = 8.0, 1.6 Hz, Py-H), 7.86 (2H, ddd, *J* = 8.5, 1.7, 0.5 Hz, Ph-H), 8.26 (1H, dd, *J* = 1.6, 0.5 Hz, Py-H); 13C NMR (75 MHz, DMSO) *δ*: 17.6, 39.2, 39.7, 49.8, 60.8, 115.0, 122.0, 127.8, 132.5, 134.0, 137.3, 152.0,152.8, 158.6, 164.3, 169.0, 175.6; HR-ESI-MS m/z: calcd for C_19_H_19_N_3_O_4_S｛(M + H)^+^｝ 385.1095, found 385.4382; Anal. calcd for C_19_H_19_N_3_O_4_S: C, 59.21; H, 4.97; N, 10.90; O, 16.60; S, 8.32; found: C, 59.23; H, 4.96; N, 10.90; O, 16.60; S, 8.31%.

*N*-(2-((6-((2,4-dioxothiazolidin-5-yl) methyl) pyridin-3-yl)oxy) ethyl)-5-methylpicolinamide (**6h**): white powder, yield 73.1%, m.p. 189–191 °C; ^1^H NMR (300 MHz, DMSO) *δ*: 2.26 (3H, s, –CH_3_), 3.12 (2H, d, *J* = 6.3 Hz, –CH_2_–), 3.49 (2H, t, *J* = 6.9 Hz, –CH_2_–), 4.18 (2H, t, *J* = 6.9 Hz, –CH_2_–), 4.42 (1H, t, *J* = 6.3 Hz, –CH–), 7.01 (1H, dd, *J* = 8.0, 0.5 Hz, Py-H), 7.35 (1H, dd, *J* = 8.0, 1.6 Hz, Py-H), 7.86–7.94 (2H, 7.92 (dd, *J* = 8.2, 0.6 Hz, Py-H), 7.91 (dd, *J* = 8.2, 2.0 Hz, Py-H)), 8.26 (1H, dd, *J* = 1.6, 0.5 Hz, Py-H), 8.46 (1H, dd, *J* = 2.0, 0.6 Hz, Py-H); 13C NMR (75 MHz, DMSO) *δ*: 17.6, 30.4, 39.2, 49.8, 60.8, 122.0, 122.2, 129.7, 132.5, 137.2, 142.6, 151.4, 152.0, 152.6 160.4, 164.5, 169.0, 175.6; HR-ESI-MS m/z: calcd for C_18_H_18_N4O_4_S｛(M + H)^+^｝386.1048, found 386.4261; Anal. calcd for C_18_H_18_N4O_4_S: C, 55.95; H, 4.70; N, 14.50; O, 16.56; S, 8.30; found: C, 55.93; H, 4.71; N, 14.51; O, 16.55; S, 8.31%.

*N*-(2-(4-((2,4-dioxothiazolidin-5-yl) methyl) phenoxy) ethyl)-4-hydroxybenzamide (**6i**): white powder, yield 80.3%, m.p. 192–194 °C; ^1^H NMR (300 MHz, DMSO) *δ*: 2.87(2H, d, *J* = 6.1 Hz, –CH_2_–), 3.47 (2H, t, *J* = 7.2 Hz, –CH_2_–), 4.12 (2H, t, *J* = 7.2 Hz, –CH_2_–), 4.45 (1H, t, *J* = 6.1 Hz, –CH–), 6.97–7.03 (4H, 6.99 (ddd, *J* = 8.8, 1.8, 0.5 Hz, Ph-H), 7.01 (ddd, *J* = 8.6, 1.1, 0.4 Hz, Ph-H)), 7.12 (2H, ddd, *J* = 8.8, 1.0, 0.5 Hz, Ph-H), 7.98 (2H, ddd, *J* = 8.6, 1.7, 0.4 Hz, Ph-H); 13C NMR (75 MHz, DMSO) *δ*: 39.2, 39.7, 49.8, 60.8, 115.0, 115.7 127.5, 127.9, 133.0,134.1, 157.7, 158.6, 168.3,169.0, 175.7; HR-ESI-MS m/z: calcd for C_19_H_18_N_2_O_5_S｛(M + H)^+^｝ 386.0933, found 386.4221; Anal. calcd for C_19_H_18_N_2_O_5_S: C, 59.06; H, 4.70; N, 7.25; O, 20.70; S, 8.30; found: C, 59.04; H, 4.71; N, 7.26; O, 20.71; S, 8.29%.

*N*-(2-((6-((2,4-dioxothiazolidin-5-yl) methyl) pyridin-3-yl)oxy) ethyl)-4-hydroxybenzamide (**6j**): white powder, yield 79.1%, m.p. 197–199 °C; ^1^H NMR (300 MHz, DMSO) *δ*: 3.12(2H, d, *J* = 6.3 Hz, –CH_2_–), 3.47 (2H, t, *J* = 6.5 Hz, –CH_2_–), 4.18 (2H, t, *J* = 6.5 Hz, –CH_2_–), 4.42 (1H, t, *J* = 6.3 Hz, –CH–), 6.99 (1H, dd, *J* = 8.0, 0.5 Hz, Py-H), 7.01 (2H, ddd, *J* = 8.6, 1.1, 0.4 Hz, Ph-H), 7.35 (1H, dd, *J* = 8.0, 1.6 Hz, Py-H), 7.98 (2H, ddd, *J* = 8.6, 1.7, 0.4 Hz, Ph-H), 8.27 (1H, dd, *J* = 1.6, 0.5 Hz, Py-H); 13C NMR (75 MHz, DMSO) *δ*: 30.5, 39.2, 49.8, 60.8, 115.6, 122.0, 127.6, 129.7, 133.0,142.6, 151.6, 157.9, 160.4, 168.3, 169.0, 175,7; HR-ESI-MS m/z: calcd for C_18_H_17_N_3_O_5_S｛(M + H)^+^｝387.0887, found 387.4101; Anal. calcd for C_18_H_17_N_3_O_5_S: C, 55.81; H, 4.42; N, 10.85; O, 20.65; S, 8.28; found: C, 55.83; H, 4.41; N, 10.84; O, 20.665; S, 8.27%.

*N*-(2-(4-((2,4-dioxothiazolidin-5-yl) methyl) phenoxy) ethyl)-5-hydroxypicolinamide (**6k**): white powder, yield 77.2%, m.p. 201–203 °C; ^1^H NMR (300 MHz, DMSO) *δ*: 2.88(2H, d, *J* = 6.1 Hz, –CH_2_–), 3.48 (2H, t, *J* = 7.1 Hz, –CH_2_–), 4.17 (2H, t, *J* = 7.1 Hz, –CH_2_–), 4.46 (1H, t, *J* = 6.1 Hz, –CH–), 7.01 (2H, ddd, *J* = 8.8, 1.8, 0.5 Hz, Ph-H), 7.10 (2H, ddd, *J* = 8.8, 1.0, 0.5 Hz, Ph-H), 7.71 (1H, dd, *J* = 8.1, 1.8 Hz, Py-H), 7.87 (1H, dd, *J* = 8.1, 0.5 Hz, Py-H), 8.46 (1H, dd, *J* = 1.8, 0.5 Hz, Py-H); 13C NMR (75 MHz, DMSO) *δ*: 39.2, 39.7, 49.8, 60.8, 114.7, 115.1, 122.0, 127.9, 134.0, 135.3, 151.3, 151.9, 158.6, 164.3, 169.0, 175.6; HR-ESI-MS m/z: calcd for C_18_H_17_N_3_O_5_S｛(M + H)^+^｝387.0887, found 387.4101; Anal. calcd for C_18_H_17_N_3_O_5_S: C, 55.81; H, 4.42; N, 10.85; O, 20.65; S, 8.28; found: C, 55.83; H, 4.41; N, 10.84; O, 20.665; S, 8.27%.

*N*-(2-((6-((2,4-dioxothiazolidin-5-yl) methyl) pyridin-3-yl) oxy) ethyl)-5-hydroxypicolinamide (**6l**): white powder, yield 70.6%, m.p. 211–213 °C; ^1^H NMR (300 MHz, DMSO) *δ*: 3.11 (2H, d, *J* = 6.3 Hz, –CH_2_–), 3.47 (2H, t, *J* = 6.9 Hz, –CH_2_–), 4.18 (2H, t, *J* = 6.9 Hz, –CH_2_–), 4.42 (1H, t, *J* = 6.3 Hz, –CH–), 7.00 (1H, dd, *J* = 8.0, 0.5 Hz, Py-H), 7.35 (1H, dd, *J* = 8.0, 1.6 Hz, Py-H), 7.70 (1H, dd, *J* = 8.1, 1.8 Hz, Py-H), 7.88 (1H, dd, *J* = 8.1, 0.5 Hz, Py-H), 8.27 (1H, dd, *J* = 1.6, 0.5 Hz, Py-H), 8.46 (1H, dd, *J* = 1.8, 0.5 Hz, Py-H); 13C NMR (75 MHz, DMSO) *δ*: 30.4, 39.2, 49.8, 60.8, 114.8, 119.8,122.1, 129.0, 135.2, 142.6, 151.3, 151.5, 151.9, 160.2, 164.3, 169.0,175.6; HR-ESI-MS m/z: calcd for C_17_H_16_N_4_O_5_S｛(M + H)^+^｝387.0887, found 387.4101; Anal. calcd for C_17_H_16_N_4_O_5_S: C, 52.57; H, 4.15; N, 14.43; O, 20.60; S, 8.25; found: C, 52.59; H, 4.15; N, 14.45; O, 20.61; S, 8.24%.

### 4.2. Biological section

#### 4.2.1. Screening of biological activity of compounds *in vitro*

According to the literature^60^, the cells containing PPAR (α, β and γ) were obtained and transfected into 96-well plates the day before for cells culture. Transfection was performed according to the instructions of human PPAR enzyme-linked immunoassay. Various compounds (**6a**–**6l**) were dissolved in DMSO and were added after the cells adhered to the wall. The Rosiglitazone was used as the positive control group and DMSO was used as the blank control group during the experiment. After 24 h, the drugs were added, and 20 μl of MTT was added to each well for another 6 h, and the cell liquid was collected by removing the culture medium, and 150 μl of DMSO was added to each hole. And the absorbance value (A) of each hole was measured under the wavelength of 460 nm. The concentration for 50% of maximal effect (EC_50_, the concentration that can cause the maximum effect of 50%) as an indicator of *in vitro* screening. EC_50_ was one of the drugs’ safety indicators, and, in general, the EC_50_ values were higher and the drug was safer.

#### 4.2.2. In vivo animal study

(1) Glucose tolerance test: The healthy mice (20 ± 2 g) were selected. After 1 week of acclimation, mice with no body surface damage, good general condition and flexible reaction were selected for the experiment. The test compounds were dissolved with DMSO, and were given a dose volume of 0.2 ml 10 g^−1^, and a dose of 10 mg kg^−1^, and given by gavage (oral) once a day. The selected mice were randomly divided into 10, each male and female, with Rosiglitazone as the positive control group and DMSO as the blank group. The mice were given the drug for 10 d, and after the last dose, the mice fasted for 12 h. The next day, blood was collected from the tail vein of the mice before and after the sugar load for 1 h and 2 h, and the blood glucose level of the mice before and after the sugar load was measured by the glucometer. (2) Insulin tolerance test: The healthy mice were randomly divided into 10, each male and female, with Rosiglitazone as the positive control group and DMSO as the blank group, given the drug continuously for 15 d. The 12 h of fasting after the last administration, blood was taken from the orbital venous plexus (used to measure the blood glucose level after fasting). Each group was immediately given a subcutaneous injection of 0.2 μg kg^−1^ of insulin. After 1 h and 2 h of injection, blood was taken again. (3) Cell survival experiment: The HUVEC cells were inoculated on the 96-well plate at the initial concentration of 3 × 10^3^ cells/holes, and 100 μl RPMI 1640 medium were added to each hole. At 5% CO_2_ and 37 °C cell cultivation in 24 h, every pore solution in different concentrations. The six replicas were performed at each concentration, with Rosiglitazone as the positive control group. And determine the hole under 460 nm wavelength absorbance value (A).
